# Osteogenic Differentiation of MSC through Calcium Signaling Activation: Transcriptomics and Functional Analysis

**DOI:** 10.1371/journal.pone.0148173

**Published:** 2016-02-01

**Authors:** Federica Viti, Martina Landini, Alessandra Mezzelani, Loredana Petecchia, Luciano Milanesi, Silvia Scaglione

**Affiliations:** 1 Institute of Biophysics, National Research Council, Genoa, Italy; 2 Institute of Biomedical Technologies, National Research Council, Segrate (Mi), Italy; 3 Institute of Electronics, Computer and Telecommunication Engineering, National Research Council, Genoa, Italy; 4 Advanced Biotechnology Center (CBA), Genoa, Italy; The University of Adelaide, AUSTRALIA

## Abstract

The culture of progenitor mesenchymal stem cells (MSC) onto osteoconductive materials to induce a proper osteogenic differentiation and mineralized matrix regeneration represents a promising and widely diffused experimental approach for tissue-engineering (TE) applications in orthopaedics. Among modern biomaterials, calcium phosphates represent the best bone substitutes, due to their chemical features emulating the mineral phase of bone tissue. Although many studies on stem cells differentiation mechanisms have been performed involving calcium-based scaffolds, results often focus on highlighting production of in vitro bone matrix markers and *in vivo* tissue ingrowth, while information related to the biomolecular mechanisms involved in the early cellular calcium-mediated differentiation is not well elucidated yet. Genetic programs for osteogenesis have been just partially deciphered, and the description of the different molecules and pathways operative in these differentiations is far from complete, as well as the activity of calcium in this process. The present work aims to shed light on the involvement of extracellular calcium in MSC differentiation: a better understanding of the early stage osteogenic differentiation program of MSC seeded on calcium-based biomaterials is required in order to develop optimal strategies to promote osteogenesis through the use of new generation osteoconductive scaffolds. A wide spectrum of analysis has been performed on time-dependent series: gene expression profiles are obtained from samples (MSC seeded on calcium-based scaffolds), together with related microRNAs expression and *in vivo* functional validation. On this basis, and relying on literature knowledge, hypotheses are made on the biomolecular players activated by the biomaterial calcium-phosphate component. Interestingly, a key role of miR-138 was highlighted, whose inhibition markedly increases osteogenic differentiation in vitro and enhance ectopic bone formation *in vivo*. Moreover, there is evidence that Ca-P substrate triggers osteogenic differentiation through genes (SMAD and RAS family) that are typically regulated during dexamethasone (DEX) induced differentiation.

## Introduction

In the last two decades, the knowledge advancement in the fields of stem cell have highlighted the possibility to harvest and manipulate in vitro adult mesenchymal stem cells derived from various tissues, including bone marrow, periostium, skeletal muscle, adipose tissue, skin and retina [1–13].

Among all stem cells reservoirs, bone marrow still remain the most commonly used worldwide, since it represents a reservoir of multi-potent mesenchymal stem cells (MSC) able to differentiate in vitro toward several lineages [3], finally offering high potential for their use in regenerative medicine applications. In particular, in order to overcome the very low frequency of occurrence among bone marrow nucleated cells, bone marrow derived MSC are typically expanded in monolayer (2D), due to their capacity to adhere to a plastic surface, before their use in combination with three-dimensional (3D) porous scaffolds. When loaded into 3D ceramic-based scaffolds the resulting constructs have been demonstrated to be osteoinductive *in vivo* [14–22]. Moreover, MSC combined with porous bioceramics have been also used with excellent results to repair large bone defects in both animal and human pilot clinical studies [17, 19, 23].

These results have opened new frontiers addressed to the investigation of biological and molecular mechanisms of MSC differentiation, and on the influence of the artificial microenvironment affecting the cellular activity [24]. Various stimuli of different nature may in fact influence MSC status, modulating their fate. Recently, mechanical and physical stimulations [25–29] have been investigated about their capability to directly induce MSC differentiation *in vitro*. Nevertheless, currently the most studied cues are represented by bio/chemical factors, which can cause signaling pathways through cell membrane receptors, second messengers and the downstream cascades, thus leading to specific cell lineages. External biochemical factors can be administered as solutions in the culture medium, or in the form of biodegradable scaffolds, where cells can be cultured and receive the released chemical components. In particular, in bone tissue engineering, the intrinsic osteoconductive properties of bioceramics work together with the biological properties of the MSC, finally triggering the formation *in vivo* of a well vascularised bone tissue. When implanted in ectopic model, MSC are able to reconstitute an *organoid* composed of both MSC derived bone tissue and bone marrow tissue originated from host hematopoietic progenitors [30].

However, one of the key elements to trigger the bone repair process is the use of a proper scaffold, that should prime cell differentiation towards the osteogenic lineage and provide the template for bone tissue formation [24]. Among modern bone substitute biomaterials, calcium phosphates represents promising alternatives as bone substitutes, mimicking the chemical composition of the natural bone tissue mineral phase [15, 18, 22, 23, 31–37]. In this system the ceramic substitute performs both as mechanical carrier, and as osteo-mimetic substrate for the MSC differentiation and new bone tissue formation [38].

Although the power of scaffolds’ morphological and topological features in affecting the cellular activity [39], biomaterial chemical composition can induce MSC osteogenic differentiation independently from the macrostructure of the substrate. This has been successfully proofed by using both bulk and porous ceramics, granules and thin scaffolds as cell substrate [30, 40].

The release of calcium (Ca^2+^) and phosphate (P) ions by dissolution is believed to be the main origin of the bioactivity of CaP biomaterials, and experimental evidence clearly indicates the key role of Ca^2+^ in osteoinduction [41, 42]. It has been measured, for example, that during bone remodeling cycle, bone resorption by osteoclasts produces local increases in the extracellular calcium concentration, reaching levels of 40mM [43], and being crucial in regulating osteoblasts proliferation, differentiation and activity. The differentiation of human bone marrow-derived MSC towards osteoblasts is accompanied by the expression of Ca^2+^ binding-proteins [44].

Although many studies on stem cells differentiation have been performed involving calcium-based scaffolds, results often focus on highlighting production of bone markers, formation and quantity of the mineralized matrix, while information about the biomolecular mechanisms involved in stem cells differentiation due to calcium cues is limited. The complex genetic programs for osteogenesis have been just partially deciphered: it is known, for example, that the master transcription factor, essential inducer of osteoblastic differentiation, is *RUNX2* [45]. Moreover, it is recognized that the main components of the bone matrix are osteocalcin, collagen, osteonectin, osteopontin, and some markers of differentiation toward osteoblast lineage (such as CRYab) have been recently identified [46]. Calcium dynamics in MSC and its role in stem cells differentiation are yet to be fully elucidated [47, 48]. The differentiation process is based on the expression of cell type-specific genes, and Ca^2+^ can play a role in controlling this through regulation of transcription factors via the action of Ca^2+^ signal transducers [47]: nevertheless, how variations in Ca^2+^ level can influence gene expression still remains unclear. Similarly, it is known that many of the cellular effects of Ca^2+^ are mediated by calmodulin, a Ca^2+^ binding protein able to bind up to 4 calcium ions leading to conformational changes which allow its binding to specific proteins to elicit a specific response [49], but the description of the different molecules and pathways operative in these differentiations is far from complete, as well as the activity of calcium in this process.

The present work aims to evaluate the involvement of extracellular calcium in MSC differentiation: a better understanding of the early stage osteogenic differentiation program of MSC is required in order to develop optimal strategies to promote osteogenesis. To this aim, different time points have been identified (i.e. 5 and 10 days) to evaluate the *short-term* osteogenic differentiation of MSC when cultured *in vitro* onto ceramic substrates. Time-dependent gene expression profiles are obtained from MSC seeded on hydroxyapatite scaffolds, together with related microRNAs (miRNA) expression. On this basis, and relying on literature knowledge, hypotheses are made on the biomolecular players triggered by the biomaterial calcium component.

Investigation of the molecular mechanisms underlying the regulation of intracellular Ca^2+^ dynamics and its correlation with the secretion of bone matrix and tissue generation may open up new directions for therapeutic strategies in bone diseases.

## Materials and Methods

### Ethics statement

Experimental animals were housed and treated in compliance with the actual national and international guidelines (Italian legislative decree 116/92, the European Community Directive 86/609 CEE and FELASA), in accordance to the authorization provided by the Italian Ministry of Health (as of D.M. 146/2009-A and subsequent integrations) and after specific ethical approval from the Ethical Committee of the National Cancer Research Institute of Genova, Italy, specific to this study and covering all experiments conducted during the study, including the euthanasia of the mice. Recipient ID 4-weeks old female mice, purchased from Charles River Italia (Charles River Lab., Calco, Milan, Italy), were kept in a controlled environment and given free access to food and water. Animals were cared for and treated according to institutional guidelines. Before the scaffold implantation, immuno-deficient mice were anesthetized by intramuscular injection of xylazine (20 microg/ml) and ketamine (30 microg/ml), to alleviate suffering during this procedure.

### Scaffold synthesis and MSC culture

A highly porous interconnected hydroxyapatite (HA) foam (ENGIpore, Finceramica Faenza, Italy) was used as substrate where culture MSC [30, 38] and induce their osteogenic differentiation. The bioceramic, according to X-ray diffraction analysis, is a single phase crystalline HA, with purity > 95%; its Ca/P ratio is 1.65±0.02 and trace elements (total heavy metals) are under the maximum allowable limit (50 mg/kg).

**Fig 1:**
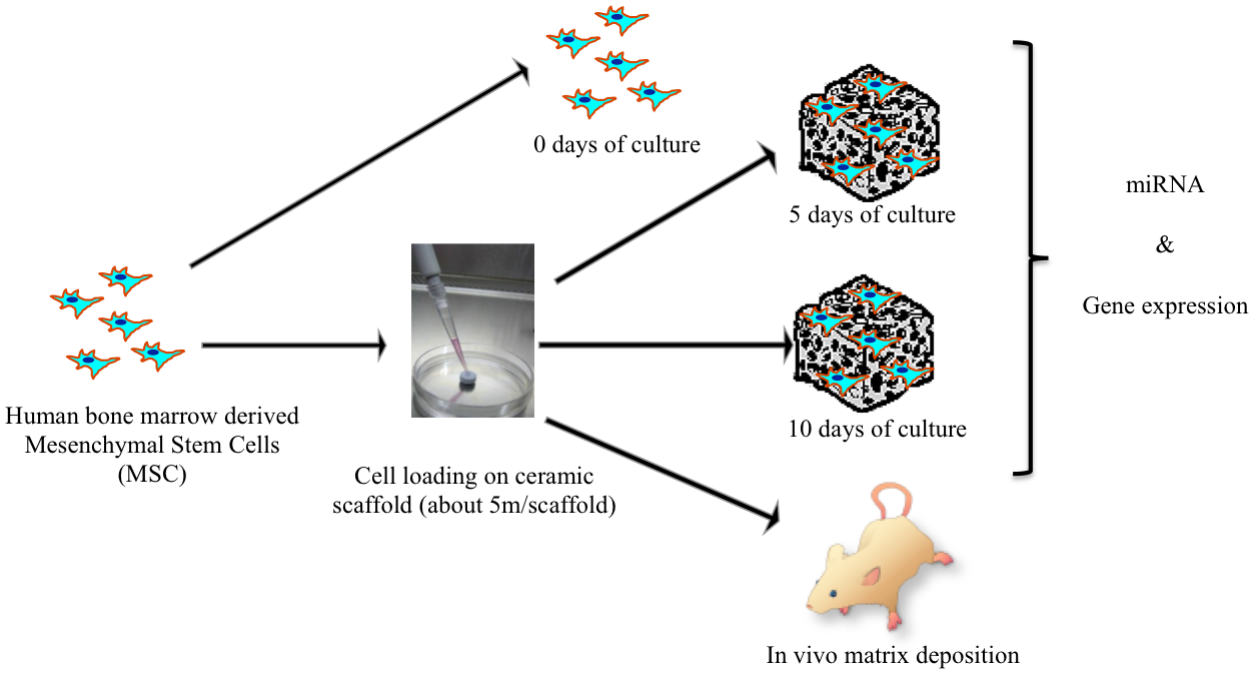
Experimental Design. Gene expression experiments were performed in time series (3 replicates for each time point: T0, T5, T10). In vivo tissue formation has been evaluated after 2-4-8 weeks from implantation of seeded biomaterial into the in vivo models (mice).

Commercially available human bone marrow mononucleated cells (Lonza, Lot. N. 081135B cat. N. 2M-125C) were obtained from the iliac crest of adult donors. Cells were expanded in Dulbecco’s modified Eagle’s medium (DMEM) enriched with 10% FCS, 100 IU/mL penicillin, and 100 mg/mL streptomycin and plated at a density of 1*10^5^ cells/cm^2^. Medium was changed twice a week. When nearly confluent, MSC were detached with 0.05% trypsin-0.01% EDTA and replated at a density of 3*10^3^ cells/cm^2^ until the next confluence. 2 million of human MSC (passage P2) were suspended in culture media and loaded into porous osteoconductive blocks (cubes of approximately 3 mm side). Sample for the *in vivo* tests were immediately implanted in mice, while samples for the *in vitro* tests were cultured *in vitro* up to 5 and 10 days respectively (Fig 1). Samples were then stored at -80°C until RNA extraction. Experiments were performed at least in triplicate.

### Experimental design

Experiments were performed in time series, considering 3 time points: cultured cells as control (T0, seeding day = 0, referred to as control, C) and cells seeded on biomaterial and analysed after 5 and 10 days as treatments (T5, seeding day = 5 and T10, seeding day = 10). Three technical replicates were produced for gene expression experiments and miRNA analysis at T5 and T10, while material extracted at T0 was used for miRNA analysis in two replicates and for gene expression study in three replicates.

### In vivo tests

Some samples (MSC-HA) were subcutaneously implanted in immuno-deficient (ID) (CD-1 nu/nu) mice following an ectopic model of bone formation [50]. To insert samples subcutaneously, skin cut of few millimeters each were performed on the back of mice, and samples implanted with surgical tweezers.

Animals were sacrificed 4–8 weeks after implantation, in agreement with previous reports [17; 30]. Four different implants were performed for each time point in different mice (two implants per animal, for a total of four mice).

### mRNA and miRNA extraction protocols

For RNA isolation, each bone matrix was transferred from -80°C storage to liquid nitrogen in a pre-chilled mortar on ice and quickly homogenized with 10 volumes of Lysis/binding buffer, avoiding any partial thawing. Homogenized samples were transferred to pre-chilled tubes and both total RNA and miRNA were extracted using the *mir*Vana^™^ miRNA Isolation Kit (Life Technologies) following manufacture’s recommendations. RNA quality and yield were first determined by NanoDrop spectophotometer and then by Agilent 2100 Bioanalyzer using Agilent RNA 6000 Nano and Agilent small-RNA Lab-Chip kits. All samples presenting RNA integrity number (RIN) equal or higher than 8 were considered suitable for the downstream applications.

Extract labelling and hybridization on chips were performed according to standard protocols compliant with Affymetrix technology.

### Expression analysis

Gene expression profile was evaluated through gene expression microarray experiments. Affymetrix GeneChip Human Gene 2.0 ST Array, a human whole-transcript coverage chip (40,716 different probes derived from RefSeq download as of February 2012), was exploited for 3 replicates for each time point. Data analysis was mainly performed in R-Bioconductor environment [51]. Oligo package was used to read HuGene2.0-st.cel format. Normalization was performed, in order to make further comparisons meaningful, through robust multi-array average (RMA) algorithm [52], implemented in Affy package. Identification of differentially expressed genes (DEG) was performed through Limma package. This analysis approach relies on two matrices: the design matrix (which provides a representation of the targets and corresponds to the microarray data matrix), and the contrast matrix (which allows to define comparisons of interest among samples). A slightly restrictive choice was made by considering just DEG presenting a log fold change (logFC) > = |2|. P value < 0.05 was set to identify significant results.

miRNA expression was evaluated through Affymetrix GeneChip miRNA4.0 Array, a small non-coding RNAs chip which interrogates all mature miRNA sequences in miRBase Release 20, together with small nucleolar RNAs (snoRNAs) and small Cajal body-specific RNA (scaRNA), for many organisms. The total amount of transcripts from all species is 30.424, while those of human origin are 6.631. miRNA analysis were performed on duplicates for T0 time point, while 3 replicates were considered for T5 and T10 time points. miRNA analysis was performed using algorithm implemented in Bioconductor analysis software, in R platform. First, miRNA expression values underwent normalization processing, in order to ensure that observed between-array differences are due to biological phenomena, excluding artifacts coming from sample handling or processing. Normalization of microRNA microarrays is problematic because of the small number of miRNA measured by the arrays, and the much smaller quantity of differentially expressed miRNA among samples, which are those suitable to be exploited for normalization. Variance stabilizing normalization (VSN) approach [53] is widely used for miRNA microarray data, since it assumes that less than half of the sequences on the arrays is differentially expressed. Therefore, vsnrma algorithm contained in vsn package has been exploited for normalization. The identification of differentially expressed miRNA was performed using Limma package: a linear model is fitted for every gene by lmFit function, and Empirical Bayes moderation of the standard errors is done by eBayes function. Differentially expressed miRNA have been selected by considering log fold change (logFC)> |1| (equal to FC>|2|) and p value < 0.05, and by applying a false discovery rate < 0.05. Among the whole list of differentially expressed miRNA, just *Homo Sapiens* related miRNA are considered in this study.

Specific databases have been referred to functionally characterize differentially expressed mRNA and miRNA. NCBI Entrez Gene [54] and DAVID web tool [55] were exploited respectively for DEG annotation and reduction of gene lists into Gene Ontology [56] related groups. miRNA post-processing relied on miRTarBase [57], to identify experimentally validated miRNA-target interactions, and miRWalk [58], to evidence miRNA recognized involvement into bone tissue, calcium signaling pathway, and osteoblasts. Due to fact that a substantial fraction of miRNA genes appear to form clusters and are transcribed in a polycistronic transcript manner, thus being expressed at similar levels and coordinately involved in regulatory networks, MetaMirClust [59] database has been exploited to define the existence of miRNA clusters among differentially expressed miRNA lists.

Network analysis were performed through STRING web tool [60], which allows identification of direct interactions among DEG, and gPROFILER [61], which enriches DEG list by adding known protein-protein interactions, thus enabling the study of relations among DEG not explicitly emerging by means of previous networks.

Home-made developed software and MySQL 5.1.73 RDBMS [http://www.mysql.com/], accessed through command line prompt or SEQUEL-PRO interface [http://www.sequelpro.com/], supported data analysis steps.

Both mRNA expression and miRNA raw data are available in ArrayExpress database (http://www.ebi.ac.uk/arrayexpress) [62] under accession numbers respectively E-MTAB-3440 and E-MTAB-3441, thus providing needed information according to MIAME guidelines [63].

### Quantitative real time PCR for gene expression results validation

In order to validate microarray data, the expression of two genes (ITGA2 and ITGB3), that resulted differentially expressed in high-throughput analysis, were evaluated through quantitative real time-PCR (qPCR) at time T0 (C_B, C_C) and at time T5 (T5_B, T5_C). Similarly, the expression of GAPDH reference housekeeping gene was evaluated.

Briefly, after genomic DNA elimination reaction, 430 ng of total RNA was retro-transcribed by "QuantiTect Reverse Transcription Kit" (Qiagen) in a final volume of 20ml; 15ng of cDNA were amplified in duplicate using "Rotor-Gene SYBR Green PCR Kit" (Qiagen) and “Rotor-Gene Q” (Qiagen) instrument. Primers were provided by Qiagen (QuantiTect Primer Assay) for ITGA2 (QIAGEN:Cat.No.QT00086695), ITGB3 (QIAGEN:Cat.No.QT00044590), and GAPDH (QIAGEN:Cat.No.QT00079247). After a PCR initial activation step (5’ at 95°C), 40 cycles of two-steps amplification were performed, consisting in 5” denaturation at 95°C followed by combined annealing/extension for 10” at 60°C. Melting curve analysis was performed to assess the specificity of amplification. Raw data were used for Ct average and standard deviation calculation, and for normalization to GAPDH. 2^-ΔCt^ and 2^-ΔΔCt^ methods were used to quantify relative expression of each target gene. To highlight differences in gene expression, a paired T-test was performed, considering significant p-values <0,01.

### Histology and Immunohistochemistry

Grafts were harvested and histologically processed. Briefly, samples were fixed in 4% buffered formalin for 4 h, decalcified with Osteodec (Bio Optica, Milan, Italy) at 37°C for 6 h and dehydrated in ethanol scale for a total of 6 h. Samples were then paraffin embedded, cross-sectioned (5-μm thick) at different levels, stained with both hematoxylin—eosin (H&E) staining. Bone matrix and blood vessels invasion were evaluated.

Some sections were subjected to immunohistochemical staining for intracellular collagen type I, with the Dako LSAB+ System-AP Kit (Code K0678), according to the manufacturer’s instructions. The technique used is based on the Labeled Streptavidin Biotin (LSAB) method. Briefly, samples were incubated with bovine serum (1% BSA) for 30 min, rinsed and incubated with the appropriately characterized and diluted primary antibody (SP1.D8 mouse antisheep pro-collagen type I, from Developmental Studies Hybridoma Bank, Iowa) for 1h.

This was followed, after rinsing, by sequential 15-minutes incubations with the biotinylated link antibody (biotinylated anti-rabbit, anti-mouse and anti-goat immunoglobulins in PBS) and alkaline phosphatase-labeled streptavidin. Staining was completed after 10-minutes incubation with the substrate-chromogen solution. The sections were then counterstained with hematoxylin for 1–2 minutes, rinsed with water and mounted with Acquatex.

Histological sections evaluation was performed using an upright microscope equipped with transmitted illumination and epifluorescence (Eclipse Ni-U, Nikon, Japan).

## Results

### Gene expression data

Information regarding the three time steps was considered, comparing gene expression levels in the following order: case A: T10 versus T0; case B: T5 versus T0; case C: T10 versus T5. Statistics about DEG lists is summarized in Table 1. The complete list of annotated DEG expression values is reported in S1 Table file. Further analyses have been performed on the group of known, coding and annotated genes, considering each comparison separately.

**Table 1:** Cardinality of mRNA Datasets.

Comparison	Total # DEG	# Annotated	# Up regulated (%)	# Down regulated
***Case A***: T10 vs T0	375	268	145 (~55%)	123
***Case B***: T5 vs T0	257	182	99 (~55%)	83
***Case C***: T10 vs T5	53	29	24 (~83%)	5

For each comparison total number of DEG is reported, together with the amount of annotated genes and the number and percentage of up and down regulated ones.

**Fig 2:**
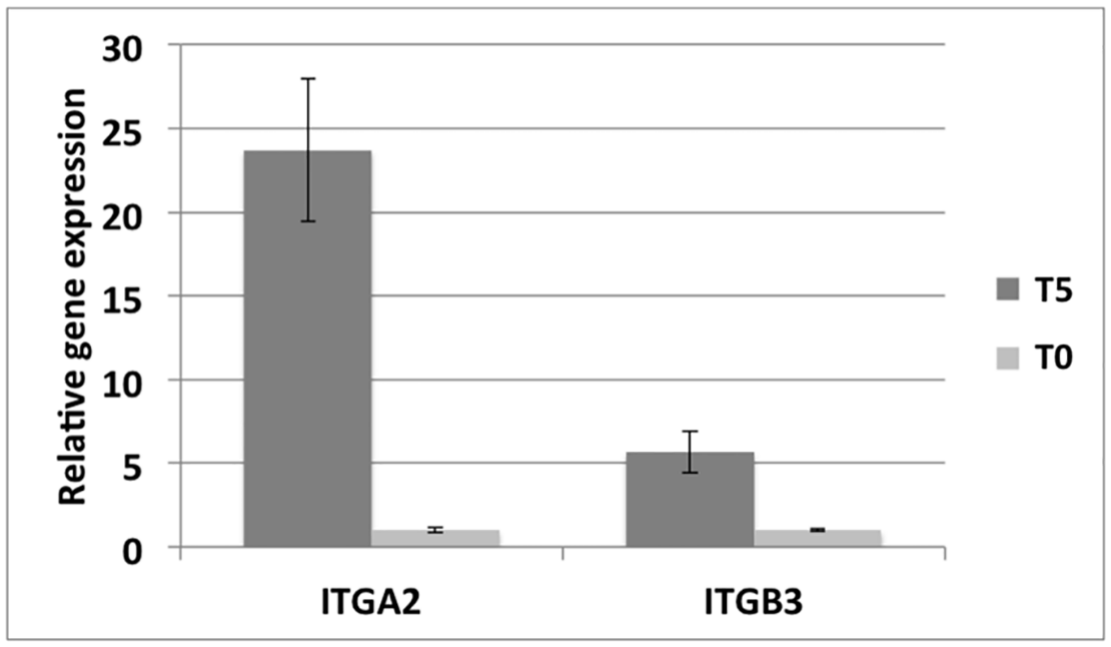
qPCR results plot. Barplot showing the average values of the relative gene expression of ITBA2 and ITGB3 genes in 2 biological replicates of T0 (gray bars) and T5 (dark gray bars) samples. Standard deviation is reported above each bar.

qPCR of two genes (ITGA2 and ITGB3) was performed for time T0 (samples C_B, C_C) and time T5 (samples T5_B, T5_C) as validation of transcriptomic data. Results are summarized in Table 2 and plotted in Fig 2. They show both ITGA2 and ITGB3 genes are over-expressed at time T5 vs time T0 (ITGA2: 4,66; ITGB3: 2,57), thus confirming values from gene-expression microarray analysis for the same comparison (ITGA2: 4,08; ITGB3: 2,57).

**Table 2:** qPCR results and statistics.

Genes	T0 samples average		T5 samples average		ΔΔCt
	Ct	ΔCt	Ct	ΔCt	
**GAPDH**	15,09		16,05		
**ITGA2**	28,21	13,12	24,51	8,46	4,66
**ITGB3**	25,28	10,19	23,67	7,62	2,57

Results of qPCR experiments for the two selected genes and the considered housekeeping gene. Threshold cycle (Ct) is reported for all genes in T0 and T5 conditions. ΔCt is calculated for both genes in comparison to the housekeeping gene. ΔΔCt indicates differential expression among the two experimental conditions.

**Fig 3:**
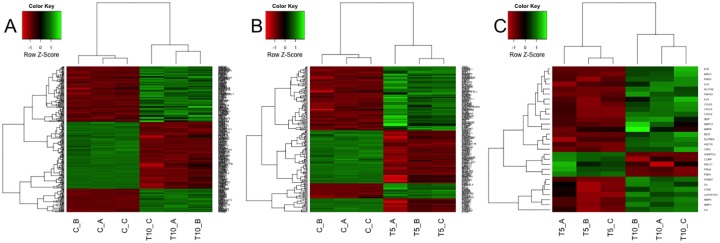
mRNA Heatmaps. Heatmaps of differentially expressed genes, obtained using logFC = 2 and pValue<0.05 thresholds, measured for cases A, B and C.

Profile of differentially expressed genes in each sample, for each comparison, is reported in Fig 3, where: heatmap A describes gene expression levels for samples compared in ‘Case A’ (3 replicates for T0 and 3 replicates for T10), heatmap B describes samples compared in ‘Case B’ (3 replicates for T0 and 3 replicates for T5), while heatmap C describes samples compared in ‘Case C’ (3 replicates for T10 and 3 replicates for T5). On top and on left side of each heatmap 2 dendrograms appear, which cluster, respectively, samples and genes relying on Euclidean distance as distance metric. It clearly emerges, both from heatmaps’ colors and from dendrograms’ distances, that replicates cluster together, thus meaning a concordant behavior, while expression profiles of DEG among diverse time points are clearly different.

**Fig 4:**
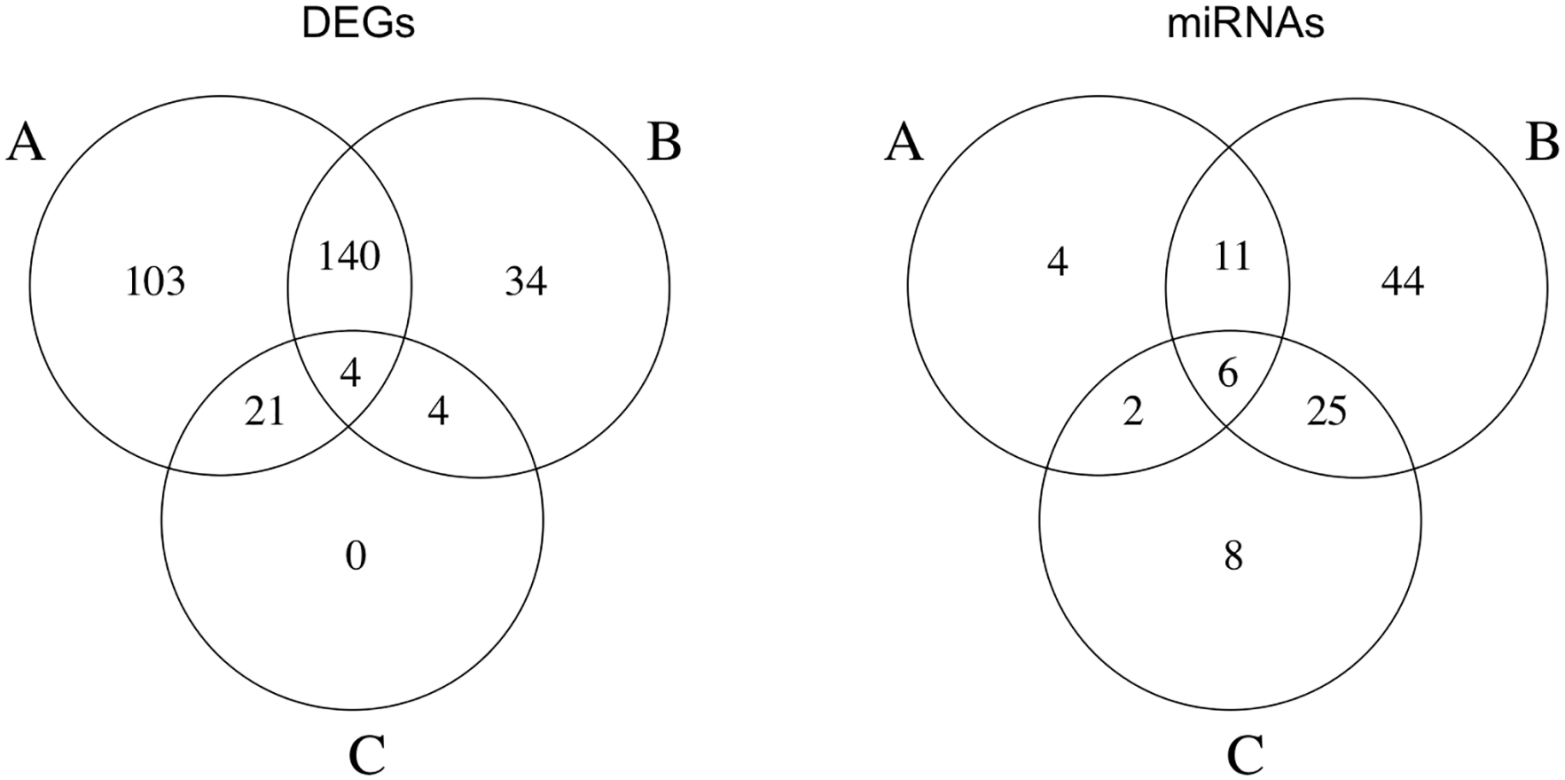
Co-occurrence of differential expression. Venn diagram of co-occurrence of differentially expressed genes (left) and miRNA (right) in cases A, B, C.

In order to define the influence of time on expression variations, a Venn diagram is presented (Fig 4, left), showing co-occurrence of DEG in considered cases: list of DEG common between the considered comparisons are reported in S2 Table file.

DEG annotation was performed separately for up- and down-regulated genes, relying on NCBI Entrez Gene information, to evidence the most involved processes during MSC differentiation toward bone. A significant proportion of DEG intervenes into bone tissue formation related mechanisms: among them calcium-based processes, bone development and pathologies, cell differentiation, extracellular matrix interaction (Table 3). Inflammatory response is also present, as a natural consequence of cell seeding on material. The complete list of genes referring to Table 3 is reported in S3 Table file.

**Table 3:** DEG Process Analysis.

Involvement	*Case A*	*Case B*	*Case C*
Bone tissue development or pathology onset	(18up+7down) ~9%	(7up+9down) ~9%	(7up) ~24%
Calcium aspects	(8up+8down) ~6%	(5up+5 down) ~6%	(1up+1down) ~7%
Cell differentiation	(8up+4down) ~4%	(3up+4down) ~4%	(2up) ~7%
Extracellular matrix interaction	(11up+9down) ~8%	(9up+11down) ~11%	(5up+1down) ~21%
Inflammatory response	(8up+1down) ~3%	(6up+1down) ~4%	(2up) ~7%

Identification of DEG’ mostly related biological process (considered for each comparison). The complete list of DEG was manually screened relying on Entrez Gene annotations: the most recurrent biological processes were highlighted and genes involved in them were counted in order to obtain statistics.

Evaluations are confirmed by analysis of gene expression overrepresentation on GO-BP and GO-MF annotations, according to DAVID Functional Annotation tool (considering medium classification stringency and p-value <0.05). One meaningful term for each cluster has been extracted and reported in Table 4.

**Table 4:** Functional Annotation Clusters.

Comparison	Enrichment score
***Case A***	
Inflammatory response	9.15
Cell differentiation	7.54
Bone development	7.51
Collagen fibril organization	7.00
Calcium ion binding	2.58
***Case B***	
Bone development	5.38
Cell differentiation	5.16
Cell adhesion	4.29
Inflammatory response	4.28
Response to calcium ion	3.00
***Case C***	
Inflammatory response	2.93
Cell differentiation	2.46
Bone development	1.73
Calcium ion binding	1.73

Functional annotation clusters related to bone formation have been identified for each comparison. Each cluster is presented with its group enrichment score, which ranks the biological significance of gene groups relying on the geometric mean of EASE scores [64] of terms involved in this cluster.

**Fig 5:**
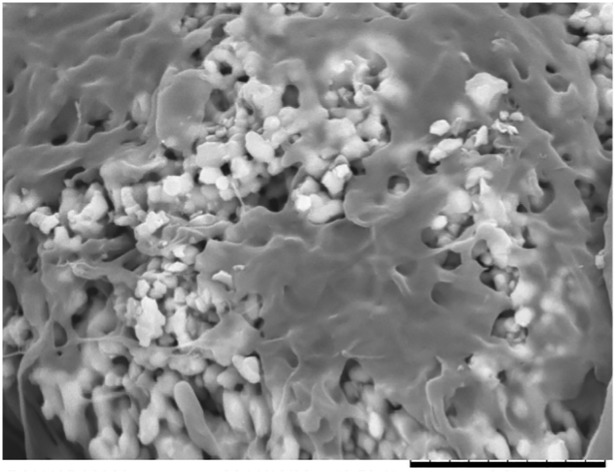
Cell adhesion. SEM image representing mesenchymal stem cells adhering to the HA surface. Bar is 10 micron.

Among those up-regulated at early time points, there are genes belonging to the family of Integrin (ITGA2: 4,08 fold change T5 vs T0; ITGB3: 2,30 fold change T5 vs T0), one of the major classes of receptors within the extracellular matrix, modulating cell-substrate interactions. Cell adhesion onto the HA scaffolds was revealed by SEM analysis, that showed spreaded cells facing the ceramic surface (Fig 5). In particular, integrins provide essential links between the extracellular environment and the intracellular signaling pathways, which can play roles in cell behaviors such as apoptosis, differentiation, survival, and transcription.

Other up-regulated genes were found belonging to classes involved in transmembrane transport and calcium metabolism, suggesting the involvement of metabolic pathways related to calcium transport from the extracellular matrix to the inner of cells (Table 5).

**Table 5:** Up/down-regulated genes grouped into main biological processes.

Transmembrane transporter	T5 vs. T0	T10 vs. T0	T10 vs. T5
SLC16A6	6,05	6,40	
SLC7A2		3,84	2,13
**Ca++ metabolism**			
STC1	5,21	6,29	
SPON1	3,26	3,77	
AREG	3,21	2,35	
PLA2G4A	2,66	3,38	
**Osteogenic differentiation**			
SPP1(OPN)	3,07	4,16	
BMP2	4,59	5,04	
IBSP (BSP)		5,92	4,07
PTHLH	2,21	3,33	
BMP6	3,57	3,54	
**Condrogenic differentiation**			
COMP		-2,30	-2,32
ACAN	-2,09	-2,80	
PRG4	4,82		-3,12
ASPN	-2,96	-3,31	

DEG evidenced for their involvement into bone-related processes.

Interestingly, as soon MSC were loaded onto the osteoconductive surface of HA an increase of several genes related to the osteogenic differentiation was observed already in the first 5 days, while the down-regulation of genes involved in chondrogenic differentiation was better shown in a period of 10 days (Table 5).

### miRNA expression data

Time series analysis was possible even for miRNA lists. The three available time steps were named, resembling gene expression, case A: T10 versus T0; case B: T5 versus T0; case C: T10 versus T5. Table 6 shows sets cardinality, while the complete list of human differentially expressed miRNA values is reported in S4 Table file.

**Table 6:** Cardinality of miRNA Datasets.

Comparison	Total # DE miRNA	# Up regulated	# Down regulated
***Case A***: T10 vs T0	23	16	7
***Case B***: T5 vs T0	86	85	1
***Case C***: T10 vs T5	41	1	40

For each comparison total number of differentially expressed miRNA is reported, together with the amount of annotated miRNA and the number and percentage of up and down regulated ones.

**Fig 6:**
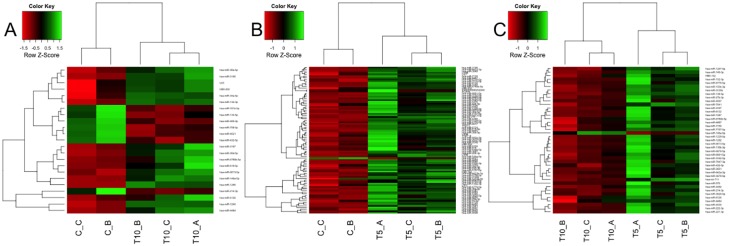
miRNA Heatmaps. Heatmaps of differentially expressed miRNA, obtained using logFC = 1 and pValue<0.05 thresholds, measured for cases A, B and C.

Heatmaps of differentially expressed miRNA in single samples for the 3 comparisons have been produced (Fig 6), considering 2 replicates available at time T0, and 3 replicates for T5 and T10 points. Heatmap A describes gene expression levels for samples compared in ‘Case A’ (2 replicates for T0 and 3 replicates for T10), heatmap B describes samples compared in ‘Case B’ (2 replicates for T0 and 3 replicates for T5), while heatmap C describes samples compared in ‘Case C’ (3 replicates for T5 and 3 replicates for T10). On top and on left side of each heatmap 2 dendrograms appear, which cluster, respectively, samples and miRNA relying on Euclidean distance as distance metric. As revealed for DEG, it emerges that replicates cluster together, thus meaning a concordant behavior, while expression profiles of selected miRNA among diverse time points are different.

Analogously to DEG’ analysis, even for miRNA a Venn diagram was produced (Fig 4, right), in order to help defining dependence of expression profiles on time. The complete list of miRNA showing differential expression in more than one comparison is reported in S2 Table file.

From cluster analysis, two miRNA clusters have been identified among differentially expressed miRNA (defined as miRNA whose chromosomal position is minor than 1000 kb): in case B an up-regulated cluster is present on chromosome 21 (has-miR-3687, has-miR-3648), while in case C a down-regulated cluster appears on chromosome X (hsa-miR-222-3p, has-miR-221-3p).

The whole lists of miRNA have been analysed through miRTarBase to identify validated targets: among them, some DEG have been identified, as reported in Table 7.

**Table 7:** Experimentally Validated microRNA-Target Interactions.

Comparisons	miRNA with validated targets	Targets in correspondent DEG lists
***Case A***	hsa-miR-146a-5p, hsa-miR-30a-5p, hsa-miR-30d-5p, hsa-miR-132-3p, hsa-miR-34a-5p, hsa-miR-409-3p, hsa-miR-134-5p, hsa-miR-708-5p, hsa-miR-181b-5p, hsa-miR-214-3p	ACSL4, ADAMTSL1, BIRC3, CALD1, CCNA2, CDCP1, CXCR4, DDAH1, FBN1, FRY, IFI44L, IL8, IRAK2, ITGA2, ITGBL1, JAG1. KIF11, LOXL1, MKI67, MMP13, NCAPG, NPR3, PLA2G4A, PTGS2, RAB27B. SLC1A5, SPP1, THBS1, TOP2A, TPM1
***Case B***	hsa-miR-30a-5p, hsa-miR-30d-5p, hsa-miR-146a-5p, hsa-miR-193b-3p, hsa-miR-29a-3p, hsa-miR-30b-5p, hsa-miR-34a-5p, hsa-miR-146b-5p, hsa-miR-320d, hsa-miR-320e, hsa-miR-16-5p, hsa-miR-130a-3p, hsa-miR-3687, hsa-miR-140-3p, hsa-miR-3648, hsa-miR-425-5p	ANKRD1, ASPM, CA12, CXCR4, DDAH1, EFNB2, IL8, IRAK2, ITGA2, ITGBL1, LMCD1, LMO7, LOXL1, NAMPT, NPR3, PDK4, PLA2G4A, PSAT1, PTGS2, RAB27B, RECK, SFRP2, SLC1A5, SLC38A5, SPP1, STC1, SULF2, THBS1, TNFAIP3, TPM1
***Case C***	hsa-miR-146a-5p, hsa-miR-138-5p, hsa-miR-193b-3p, hsa-miR-152-3p, hsa-miR-140-3p, hsa-miR-222-3p, hsa-miR-214-3p, hsa-miR-27b-3p, hsa-miR-221-3p, hsa-miR-130b-3p	IL8, MMP1, MMP13, MMP3

For each comparison, experimentally validated microRNA-target interactions have been reported, according to miRTarBase database. miRNA presenting validated targets are listed, together with validated targets corresponding to identified DEG.

Among differentially expressed miRNA, miRNA specifically related to osteoblasts, bone tissue and calcium pathway can be found. They have been reported in Table 8, according to data maintained into miRWalk database.

**Table 8:** Differentially Expressed miRNA Concerning Bone Aspects.

Comparison	miRNA in osteoblasts	miRNA in bone tissue	miRNA in calcium pathway
***Case A***	hsa-miR-1290, hsa-miR-432-5p, hsa-miR-4521	hsa-miR-146a-5p, hsa-miR-1290, hsa-miR-6126, hsa-miR-30a-5p, hsa-miR-30d-5p, hsa-miR-132-3p, hsa-miR-34a-5p, hsa-miR-432-5p, hsa-miR-4521, hsa-miR-181b-5p, hsa-miR-214-3p	hsa-miR-146a-5p, hsa-miR-1290, hsa-miR-619-5p, hsa-miR-30a-5p, hsa-miR-30d-5p, hsa-miR-132-3p, hsa-miR-34a-5p, hsa-miR-432-5p, hsa-miR-4521, hsa-miR-134-5p, hsa-miR-708-5p, hsa-miR-181b-5p, hsa-miR-214-3p
***Case B***	hsa-miR-1202,hsa-miR-1207-5p, hsa-miR-1229-5p, hsa-miR-1290, hsa-miR-140-3p, hsa-miR-16-5p, hsa-miR-204-3p, hsa-miR-4417, hsa-miR-4433-3p, hsa-miR-4492, hsa-miR-5739, hsa-miR-575	hsa-miR-1202, hsa-miR-1207-5p, hsa-miR-1229-5p, hsa-miR-1273g-3p, hsa-miR-1275, hsa-miR-1290, hsa-miR-130a-3p, hsa-miR-140-3p, hsa-miR-146a-5p, hsa-miR-146b-5p, hsa-miR-1587, hsa-miR-16-5p, hsa-miR-193b-3p, hsa-miR-204-3p, hsa-miR-29a-3p, hsa-miR-30a-5p, hsa-miR-30b-5p, hsa-miR-30d-5p, hsa-miR-34a-5p, hsa-miR-4417, hsa-miR-4433-3p, hsa-miR-4433b-3p, hsa-miR-4459, hsa-miR-4485, hsa-miR-4487, hsa-miR-4492, hsa-miR-4507, hsa-miR-4508, hsa-miR-4521, hsa-miR-4530, hsa-miR-4532, hsa-miR-5739, hsa-miR-575, hsa-miR-6126, hsa-miR-6132, hsa-miR-642a-3p	hsa-miR-1202, hsa-miR-1207-5p, hsa-miR-1273g-3p, hsa-miR-1275, hsa-miR-1290, hsa-miR-130a-3p, hsa-miR-140-3p, hsa-miR-146a-5p, hsa-miR-146b-5p, hsa-miR-16-5p, hsa-miR-193b-3p, hsa-miR-204-3p, hsa-miR-29a-3p, hsa-miR-30a-5p, hsa-miR-30b-5p, hsa-miR-30d-5p, hsa-miR-320d, hsa-miR-320e, hsa-miR-34a-5p, hsa-miR-3648, hsa-miR-3687, hsa-miR-425-5p, hsa-miR-4417, hsa-miR-4433-3p, hsa-miR-4433b-3p, hsa-miR-4459, hsa-miR-4485, hsa-miR-4487, hsa-miR-4492, hsa-miR-4507, hsa-miR-4508, hsa-miR-4521, hsa-miR-4530, hsa-miR-4532, hsa-miR-4649-5p, hsa-miR-5739, hsa-miR-575, hsa-miR-6085, hsa-miR-6124, hsa-miR-6132, hsa-miR-619-5p, hsa-miR-642a-3p
***Case C***	hsa-miR-1202, hsa-miR-1207-5p, hsa-miR-1229-5p, hsa-miR-152-3p, hsa-miR-221-3p, hsa-miR-432-5p, hsa-miR-4492	hsa-miR-1202, hsa-miR-1207-5p, hsa-miR-1229-5p, hsa-miR-130b-3p, hsa-miR-138-5p, hsa-miR-140-3p, hsa-miR-146a-5p, hsa-miR-1587, hsa-miR-193b-3p, hsa-miR-214-3p, hsa-miR-221-3p, hsa-miR-222-3p, hsa-miR-432-5p, hsa-miR-4487, hsa-miR-4492, hsa-miR-4507, hsa-miR-4530, hsa-miR-6126, hsa-miR-6132, hsa-miR-642a-3p	hsa-miR-1202, hsa-miR-1207-5p, hsa-miR-130b-3p, hsa-miR-138-5p, hsa-miR-140-3p, hsa-miR-146a-5p, hsa-miR-152-3p, hsa-miR-193b-3p, hsa-miR-214-3p, hsa-miR-221-3p, hsa-miR-222-3p, hsa-miR-27b-3p, hsa-miR-432-5p, hsa-miR-4487, hsa-miR-4492, hsa-miR-4507, hsa-miR-4530, hsa-miR-6132, hsa-miR-642a-3p

For each comparison, differentially expressed miRNA concerning bone tissue related processes are reported, according to data maintained into miRWalk database.

### Gene networks

For each considered condition (cases A, B, C), gene networks of DEG have been reconstructed, where nodes represent genes, and edges their interactions reported in literature. Networks elements, provided by STRING web tool for the 3 comparisons, are shown in S5 Table. A wide subset of DEG presents interactions, meaning a close functional relation among the differentially co-expressed genes. In particular, genes related to bone tissue formation are connected together within the same network. Examples are: BMP2, which clusters with COL1A2, COL1A1, IBSP, ACAN, SPP1 in network of case A; matrix metallopeptidases (MMP1, MMP10, MMP3), which are associated in case B network; interleukins (IL33, IL6, IL8) and chemokines (CXCL5, CXCL6), which group together in network of case C.

gProfiler allowed to significantly enriching DEG network by means of BioGRID knowledge about protein-protein interactions, thus highlighting indirect relations among proteins. Row data are reported in S6 Table, which shows both the original lists of DEG (‘Gene names and descriptions’ list) and the additional genes representing PPI terms (‘neighbors’ list). Relations between PPI terms and DEG are shown in ‘graph structure’ list. gProfiler enrichment allowed either highlighting aspects not revealed by DEG set, or further underlining crucial issues. For example, classifying data from case A on Gene Ontology Biological Processes, it emerges that 34 newly added genes (over 984) are related to cell-cell or cell-matrix adhesion (GO:0022610), underlying importance of this process in bone formation. Analogously, analyzing enrichment genes in all comparisons the developmental and the immune system processes arise as crucial (Table 9).

**Table 9:** Involvement of enrichment genes into developmental and immune system processes.

**Developmental process (GO:0032502)**		
**Comparison**	**# genes**	**% of gene hit against total # genes**
***Case A***	169	17.2
***Case B***	95	21.3
***Case C***	27	30.3
**Immune system process (GO:0002376)**		
**Comparison**	**# genes**	**% of gene hit against total # genes**
***Case A***	99	10.1
***Case B***	66	14.8
***Case C***	18	20.2

PPI genes obtained from gProfiler were analyzed through Panther tool and functional involvement into developmental and immune system processes is reported for each comparison.

### Immunohistochemistry and histological data analysis

**Fig 7:**
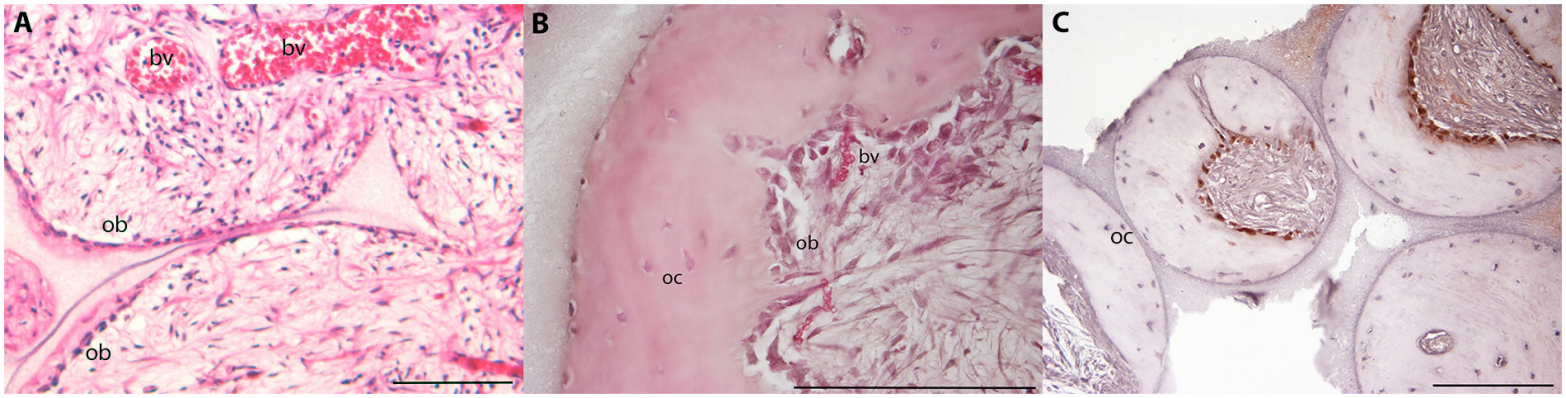
Haematoxylin-eosin staining and IHC. HE analyses have been performed on samples harvested after 2 (panel A) and 8 weeks (panel B) of implantations in in-vivo models, reporting different levels of bone matrix deposition. Ob = osteoblasts, oc = osteocytes, bv = blood vessels. Panel C reports IHC analyses performed after 8 weeks of implantation. IHC expresses intracellular pro-collagen coloured in red, while blue cells (i.e. negative) were obtained after counterstaining with haematoxylin. Bars are 50 micron.

The histological analysis of HA samples implanted in vivo highlighted the MSC adherent to the external osteoconductive surface starting their differentiation and neobone tissue deposition already 2 weeks after implantation, while host tissue started to colonize the cavities of the implant (Fig 7, panel A); an incoming vascularization was also evident. A marked bone matrix deposition was observed after 8 weeks of implantation (Fig 7, panel B), with osteoblasts and osteocytes well visible within the newly formed bone tissue. In particular, at the interface between the HA and the neobone several osteocytes are well visible interaction with the osteoconductive surface of HA.

Expression of intracellular pro-collagen protein was investigated through immunohistochemistry by staining constructs harvested after 8 weeks of implantation (Fig 7 panel C). Osteoblasts lining the interface between the newly formed bone tissue and the connective tissue filling the porosities of HA were all positive to COLI, while osteocytes already embedded in their lacunae within the bone matrix were negative to the staining, demonstrating the different maturation of cells during their osteogenic differentiation.

## Discussion

The combination of calcium phosphate (CaP)-containing biomaterials and adult progenitor cells has been indicated as a promising tissue engineered solution for skeletal regeneration. The identification of signalling cascades that are triggered in progenitor cells by CaP based scaffolds is an essential step in understanding the mechanism of cellular activation/differentiation that culminates in bone formation *in vivo*. Although much research has been conducted on cell differentiation to develop cell/biomaterial based tissue engineered products, the endogenous mechanism offered by CaPs on the progenitor populations has been largely neglected. Moreover, the biochemical mechanisms triggering the regulation of osteogenic markers *in vitro* remain typically untested in vivo, through a proper validation.

In this paper we evaluate different transcriptomic levels (gene expression and miRNA arrays) to investigate possible pathways involved in early calcium-phosphate-driven *in vitro* differentiation of mesenchymal stem cells. Mechanisms of osteoinduction by Ca-P *in vivo* have been also described as proof of the complete cell maturation towards the osteogenic fate.

The cross-correlation between transcriptomics and biological process analysis carried out in this work revealed the progressive differentiation of osteoprogenitor cells, when cultured onto HA scaffolds, through preosteoblasts, proliferating osteoblasts, mature osteoblasts, and osteocytes, which is associated with various expression patterns of bone-related genes, sequentially activated by osteoblast-specific transcription factors and fluctuating during the stages of cell growth (proliferation), maturation (differentiation) and, finally, mineralized matrix deposition *in vivo*.

Various analysis have been carried on about the transcription level, starting from gene and miRNA expression high-throughput data, to achieve meaningful information. After defining the lists of differential expression at various time points, similarity clusters (heatmaps and dendrograms) on the selected genes and miRNA were produced, to evaluate consistency of replicates and difference among diverse time points; Venn diagrams were plotted to show expression overlap among different analyzed conditions; functional annotation of genes and miRNA was performed, to identify active biological process, with particular interest for bone-related processes.

Late genes such as osteopontin (SPP1), bone sialoprotein (IBSP) and bone morphogenic protein (BMP) were found overexpressed over time, whereas no significant differences of gene expression were observed for alkaline phosphatase (ALP), collagen type I (COLI), and osteonectin (SPARC), whose levels increase prior to the onset of mineralization (Table 4).

Major differences in gene expression dynamics were observed during first days of cell-substrate interaction, since no DEG is peculiar of C set (i.e. T10 vs. T5); such differences were associated with activation of molecular signaling pathways related to bone remodeling, as confirmed by SPP1(ON) and BMP family gene levels. BMPs are members of the transforming growth factor-b (TGF-b) family that bind to type II and type I serine-threonine kinase receptors, and transduce signals through Smad and non-Smad signaling pathways. Recently, it has been shown that calcium up-regulates gene transcription of BMP2 in human MSC [65]. This finding is confirmed herein, as BMP2 and BMP6 gene expression levels were high already after few days of in vitro culture, finally supporting the key role for BMP signalling pathway in calcium phosphates driven osteogenesis.

Calcium (Ca^2+^) is an essential signaling messenger that modulates a variety of cellular functions ranging from cell growth to differentiation to cell death. On the contrary, osteopontin transcription was shown to increase in relation to phosphate, the other ion building the hydroxyapatite crystals, which acts on the OPN promoter, through the glucocorticoid receptor [66].

Osteocalcin and osteonectin genes were not significantly regulated during in vitro cell culture, although a complete cellular maturation and in vivo bone formation was observed within HA constructs. These results are however in accordance to what previously reported about the ability of osteoblasts to form bone matrix even in mice in which these genes have been previously silenced [67,68].

Interestingly, the combination of Ca^2+^ and PO^3-^_4_ ions in vitro triggers an osteogenic differentiation through BMPs/SMAD and RAS signaling pathways, involving genes typically regulated during the dexamethasone (DEX) induced differentiation [69].

Smad1/5/8 are intracellular signaling proteins that can modulate BMP-mediated osteogenesis, and the intensity of BMP signals can be determined by BMP receptors via Smad1 C-terminal phosphorylation. Moreover, non-Smad associated kinase cascades are also activated by TGF-beta superfamily members: in particular, some non-smad pathways used by BMP-2 include RAS/Raf/ERK [70].

DEX, a synthetic glucocorticoid, has been shown to induce bone marrow derived osteoprogenitor cells towards osteoblastic phenotype [71]. In particular, several genes found up-regulated during MSC culture onto HA substrates (Table 10) belong to the RAS superfamily, and their expression appears to be regulated positively by glucocorticoids, such as DEX [72].

**Table 10:** Differentially Expressed genes Concerning RAS pathway.

	T5 vs. T0	T10 vs. T0
RASD1	3,75	4,03
TMEM158	3,32	3,02
RAB27B	5,01	5,60
RASEF	2,57	3,40

DEG evidenced for their involvement into RAS pathway.

The up-regulation of SMAD and RAS family genes when MSC are cultured with osteogenic medium containing DEX is expected and consistent with the fact that the DEX regulates the osteogenesis of human MSC and mineralization in vitro. Interestingly, only by using Ca-P as substrate, these genes were equally upregulated, suggesting an osteogenic commitment of hMSC cultured on HA in basal medium, similar to the results achievable by supplementing growth factors for directing cell fate towards osteoblast lineage. Moreover, the Ca-P induced osteoblastic commitment also guaranteed the initiation and maturation of ectopic bone formation. Already 14 days after in vivo implantation, MSC induced neo bone formation appeared, confirming the key role of mineral phase as autoconsistent osteoinductive stimulus.

It is worth to note the significant involvement of inflammation players in the osteogenesis process. This is testified by the presence of a high number of inflammation-related genes among the list of DEG (S3 Table). Although mechanisms underlying their regulatory action in osteogenic process have not been completely elucidated, recent studies showed that many mediators of the inflammatory response play a key role in osteogenesis. Among DEG, a set of genes known to be involved both in inflammation and bone formation appears, which includes: IL6, encoding a cytokine that acts in the maturation of B cells, is known to be an important pro-inflammatory cytokine secreted by osteoblasts in bone resorption process [73], and in human BMSCs it seems to positively influence the mitogen-activated protein kinase signaling cascade, essential for normal skeletogenesis and bone formation [74]; the protein encoded by PTGES gene, a glutathione-dependent prostaglandin E synthase, was evidenced in the monolayered MSCs [75] and several studies suggest this gene contributing to decrease cell proliferation and induces osteogenesis [76]; IL1, an heterodimer containing α and β subunits, seems to intervene in bone remodeling process [77].

Recently, it was also demonstrated the importance of miRNA, key post-transcriptional regulators of gene expression, in the control of osteoblast and osteoclast differentiation and function. Various positive and negative miRNA regulators of bone remodelling have been identified, and in some cases used for therapeutic purpose [78].

Several studies have focused on miRNA modulated by BMP signaling as a means to understand the role of miRNA in osteoblasts. Since some miRNA can be co-expressed and/or co-regulated, it is possible that families of miRNA may promote one phenotype at the expense of another. Among them, inhibition of miR-138, here found, it was shown to markedly increase osteogenic differentiation in vitro and enhance ectopic bone formation in vivo [79]. Moreover, down-regulation of miR-140 and miR-193, expressed during cartilage development, was also shown, in accordance with the expressions of genes typically involved during MSC differentiation towards the chondrogenic lineage. This is in agreement with our results showing a process of *in vivo* intramembranous ossification, in spite of the endochondral one, where the bone tissue is created by cartilaginous tissue expressing specific chondrogenic genes.

## Conclusions

The presented study attempts to investigate which molecular players are activated by calcium-phosphate based scaffolds. The strategy employed herein highlights the importance of the microenvironment (i.e. in principle chemical conditioning provided by the substrate), for cell activation and subsequent tissue formation. A better knowledge of the molecular mechanisms that control growth and differentiation of MSC may enable the production of biologically functional biomaterials with defined cell-specific properties for regenerative medicine strategies. In particular, the development of composite materials, which combine the easy workability of polymers with the osteoinductive properties of Ca-P phases, allows realizing clinically relevant biomaterials, avoiding the use of specific exogenous growth factors.

## Supporting Information

S1 Table Annotated DEG expression values. The complete list of differentially expressed genes together with their expression levels (logFC) and p values.
(XLSX)

S2 Table Differentially expressed genes and miRNA common among different comparisons. List of genes and miRNA showing differential expression in more than one comparison, indicating a strong dependence of their expression on time. Genes present in all comparisons are highlighted in yellow, miRNA present in all comparisons are highlighted in green.
(XLSX)

S3 Table List of DEG involved in relevant biological processes. The complete list of DEG (considered for each comparison and separated into up- and down- regulated) intervening into bone tissue formation and related mechanisms.
(XLSX)

S4 Table Differentially expressed miRNA values. The complete list of human differentially expressed miRNA together with their expression levels (logFC) and p values.
(XLSX)

S5 Table STRING networks. Networks elements, provided by STRING web tool, are reported for the 3 comparisons of the time series (A, B, C).
(XLSX)

S6 Table gProfiler enrichment. Row data of DEG networks (comparisons A, B, C) obtained by gProfiler, which allows a significant enrichment by means of BioGRID knowledge about protein-protein interactions, thus highlighting indirect relations among proteins. Genes originally included in DEG list are reported in ‘Gene names and descriptions’ section, while genes involved into PPI and therefore added to original networks are reported in ‘graph structure’ section.
(XLSX)
